# Phase envelopes in reservoir fill analysis: Two contrasting scenarios

**DOI:** 10.1038/s41598-024-56058-6

**Published:** 2024-03-07

**Authors:** Khaled R. Arouri, Carlos G. Herrera

**Affiliations:** Saudi Aramco, 31311 Dhahran, Saudi Arabia

**Keywords:** Fossil fuels, Crude oil, Geochemistry

## Abstract

Phase envelopes are routinely employed by reservoir engineers for fluid characterisation. These envelopes are controlled by reservoir fluid composition, pressure and temperature. As a result of increasing source-rock maturation, fluids with decreasing molecular weights and densities and increasing gas-to-oil ratios (and hence different phase envelopes) are generated, which are thus linked to fluid history. In addition to their importance for exploration, charge models can play a key role in constraining reservoir models and optimising field development, particularly when pressure–volume-temperature (PVT) data are properly integrated with fluid geochemistry. Two contrasting scenarios of fluid phase evolution from two different fields are presented, and their relations to charge analysis and reservoir models are discussed. The first example discusses the identification, based on hydrocarbon geochemistry complemented by overlapping modeled phase envelopes, of compartmentalised filling cycles in what was initially considered a single oil-rimmed gas accumulation. The second example presents an opposite scenario where two wet gas accumulations 20-km apart laterally and 400-feet average depth difference appear to represent a single more-expansive accumulation spread over areas of variable PVT conditions and reservoir qualities. The wet gas across both accumulations is characterised by a continuous phase evolution pattern that shrinks systematically (cricondentherm shifts to lower temperature and cricondenbar to lower pressure), suggestive of phase fractionation of a charge of single maturity. The proposed gas distribution model represents a discovery of a hybrid conventional and unconventional (tight sand) system, with potential for basin-centered gas. These findings provided better understanding of observed and projected fluids, impacting the development and completion plans by locating new gas producers. A recent well drilled midway between the two accumulations indeed tested wet gas, confirming fluid connectivity. Future work will attempt to link the gas distribution model with seismic attributes.

## Introduction

Integrating geoscience and reservoir engineering in the oil industry presents many challenges^1^, but offers more opportunities. For example, pressure-volume-temperature (PVT) data are used routinely by reservoir engineers for fluid characterisation and reservoir simulation, mainly for production plans^2–5^, but these data have other important applications in petroleum exploration, particularly in charge history analysis and migration studies^6,7^. We recently discussed the value added by integrating PVT data and hydrocarbon geochemistry for exploration, reservoir characterisation and reservoir management^8^, and for fault and seal integrity assessment^9,10^. This paper elaborates on the original concept introduced by Di Primio et al^6^ and Mills et al^7^ on the use of PVT and phase behaviour in exploration, and applies the knowledge for reservoir fill analysis of three complex hydrocarbon accumulations in the Arabian Basin. Reservoir fill analysis maximises the value gained from existing PVT data to better understand fluid distribution, reduce uncertainty, and constrain reservoir models.

**Figure 1:**
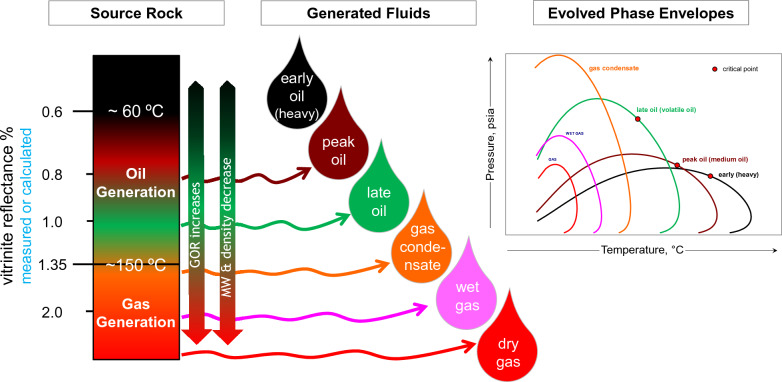
Progress of source-rock maturation results in fluids of different properties and systematic evolution of phase envelopes.

Reservoirs with overlapping phase envelopes represent different cycles of filling coming from different kitchens or from charges of differing maturities from the same kitchen. Increasing burial depth and temperature of the source rock will lead to multiple stages of hydrocarbon generation that become progressively lighter with increasing maturation, resulting in continuous or multiple cycles of reservoir filling. Figure 1 shows a basic but important concept to start linking the PVT reservoir engineering data (phase envelops) with geochemistry analysis (vitrinite reflectance). During maturation, the molecular weight and density decrease and the gas-to-oil ratio (GOR) increases, thereby controlling the evolution and behaviour of the generated fluid phase (Fig. 1). A single charge will be characterised by a single thermal maturity, while several charges of different maturities provide fluids of different maturities and compositions. Charges of higher maturities will possess higher concentrations of lighter hydrocarbons and, hence, higher GOR values.

**Figure 2:**
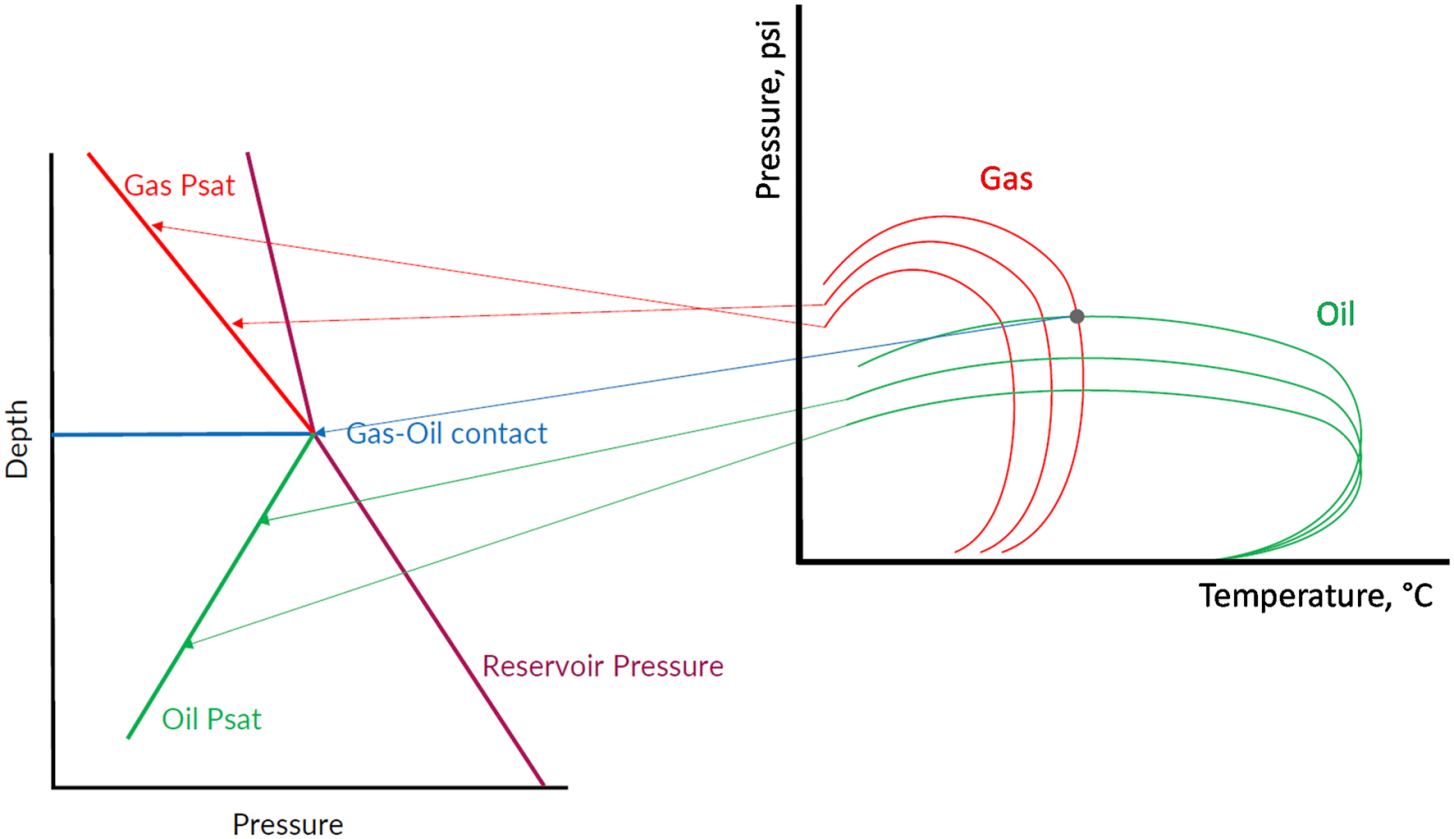
Phase envelope behaviour in a continuous oil-gas column. However, unsystematic behaviour in pressure, GOR-saturation pressure relationships, fluid composition and geochemistry can reveal compartmentalisation of different fluid systems, as illustrated in the first example.

During upward migration and changing reservoir PVT conditions, light hydrocarbons will increase, leading to reduction in density and viscosity, causing systematic shrinking in phase envelopes, hence, shifting the cricondentherm to lower temperature, and the cricondenbar to lower pressure^6,7^. The schematic in Fig. 2 illustrates the behaviour of phase envelopes in a continuous oil-rimmed gas accumulation (ORGA). The systematically shrinking gas phase envelopes and the systematically shrinking oil phase envelopes intersect at the gas-oil contact in a saturated petroleum system. While systematic depth gradients in reservoir pressures and saturation pressures are expected in a continuous reservoir, separate compartments will be characterised by offsets in pressure trends, and those will exhibit larger differences in their phase envelopes.

Two contrasting phase behaviour scenarios are discussed in this paper: (1) overlapping phase envelopes of black oil and gas condensates, and their relations to fluid geochemistry in understanding fluid distributions and possible compartmentalisation, and (2) systematic shrinking of phase envelopes of wet gas in accumulations hitherto identified as separate fields, and the possible connectivity of the two accumulations.

## Methods

Reservoir PVT data, such as the gas-oil ratio (GOR), saturation pressure (Psat), heptane-plus fraction, and iC_4_/nC_4_ were collected from in situ representative downhole or recombined wellstream composition samples. Fluid geochemical characterisation was conducted on depressurised oil and gas samples. Methods included stable carbon isotope analysis (δ^13^C, ^13^C/^12^C, reported in parts-per-thousand, ‰) for the C_1_-C_5_ hydrocarbons using an Agilent 6890 GC equipped with a 30-m Poraplot GSQ column and a Thermo GC Combustion III system interfaced to a Delta Plus stable isotope ratio mass spectrometer (reproducibility ± 0.2‰). The NBS-19 was used as the standard. Aromatic biomarkers were analysed in selected ion monitoring (SIM) mode, monitoring for key ions (m/z 178, 184, 192, 198 and 231), using gas chromatography-mass spectrometry (HP 6890 GC interfaced to an HP mass selective detector MSD 5973). Vitrinite reflectance equivalent (VRe %) was calculated using the phenanthrene and methylphenanthrene data^11^. A Peng-Robinson Equation of State was tuned for the fields using laboratory PVT samples (Constant Composition Expansion “CCE” and Constant Volume Depletion “CVD” experiments) in order to generate reliable phase envelopes.

## Examples and discussion

### Phase envelopes identify filling sequence and different fluid systems in a single field

**Figure 3:**
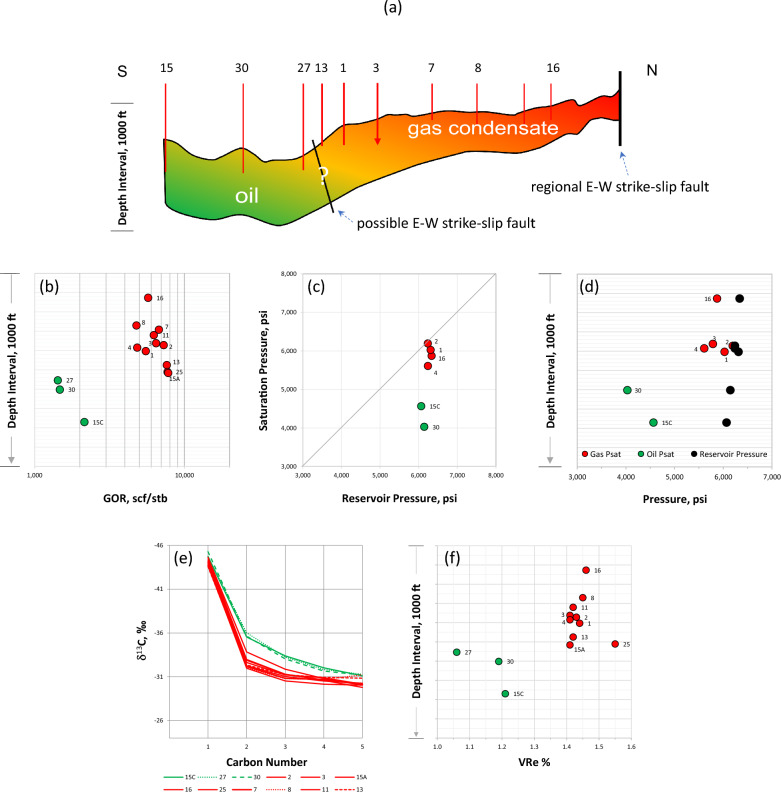
(**a**) Schematic north–south cross section illustrating the distribution of black oil (green) and gas condensate (red) in Field G. (**b**) GOR variations with depth. (**c**) Reservoir pressure versus saturation pressure. (**d**) Depth profiles for reservoir pressure and saturation pressure. (**e**) δ^13^C fingerprints for C1-C5 gas components in both oil and gas samples. (**f**) Depth gradients in vitrinite reflectance equivalent, VRe %.

During the development of the gas Field G, black oil was discovered downdip in the southern region of the field (Fig. 3a). This study deals with the three oil discoveries (15A, 27, and 30) and their relationships to the updip gas condensates in the main part of the field (rest of samples, Fig. 3a). The GOR (standard cubic feet per stock tank barrel, scf/stb) variations with depth are shown in Fig. 3b. Both oils and gas condensates are monophasic undersaturated fluids, where saturation pressures (Psat) < reservoir pressures (Fig. 3c). Based on the small differences in reservoir pressures and the fact that the observed differences in the Psat for the oils and gas condensates are within the range expected in an oil-rimmed gas accumulation (ORGA), a single tank was initially proposed. The abrupt offset in fluid composition gradient, such as GOR (Fig. 3b) and the clear offsets in the reservoir and saturation pressures for the oil wells (Fig. 3d), however, suggest that the oil and gas are not a single ORGA but constitute separate compartments.

The reservoir engineering attributes and the inferred fluid relationships discussed above were further scrutinised by inspecting the thermal maturity of both the gas range and the liquid range in each sample. Examples of the thermal maturity parameters used include the carbon isotope composition (δ^13^C) of the C1-C5 gas range (Fig. 3e) and the equivalent vitrinite reflectance VRe % (Fig. 3f) estimated by measuring the abundance of thermodynamically stable compounds to the abundance of less-stable compounds, expressed as the methylphenanthrene index MPI^11,12^. The consistency of maturity differences of light and heavier hydrocarbons in each fluid sample indicates separate charges that are distinctly different in their maturities, i.e. isotopically lighter low-maturity oil associated with low-maturity gas, and isotopically heavier gas of higher maturity (Fig. 3e,f). These geochemical data added further clarity to the reservoir engineering data in supporting the accumulation of oil and gas condensate in separate compartments rather than in a continuous reservoir.

**Figure 4:**
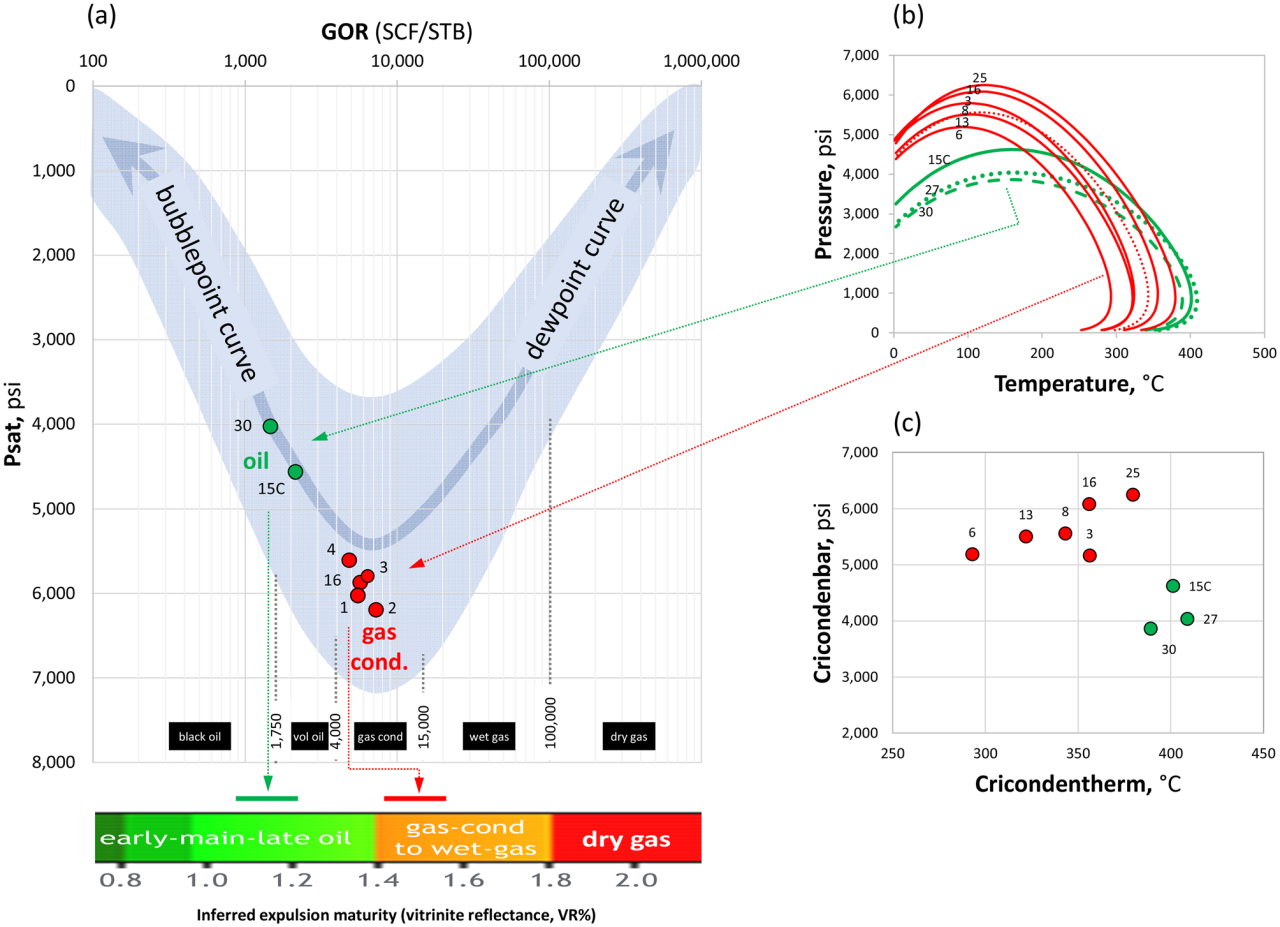
(**a**) GOR-Psat relationships for fluids from Field G (black oil in green; gas condensate in red). The blue band represents the global phase prediction template^13,14^. Inferred source-rock maturity windows for both oil (green bar) and gas condensate (red bar) are indicated. (**b**) Phase envelopes for the black oils (green) and gas condensates (red) in Field G. (**c**) Clustering of oils and gas condensates based on their cricondentherm-cricondenbar relationships.

The inferred expulsion maturity windows for the oil and the gas condensate are shown in Fig. 4a and are discussed in the framework of GOR, Psat and phase envelopes (Fig. 4b). The GOR-Psat crossplot shown in Fig. 4a compares samples with available Psat data from Field G. As the charge moves to lower pressures, the GOR splits into two trends: bubblepoint curve and dewpoint curve. As such, fill-spill migration normally leads to the accumulation of oil updip gas along the bubblepoint trend, while phase separation of a single charge within a trap will lead to successively lighter fluids in shallower sections while maintaining identical fluid maturity. Given the occurrence of oil below the level for gas condensate in Field G, the fill-spill fluid relation is not applicable here; and given the varying fluid maturities, the phase separation relation is also not applicable. The lower values for both parameters (GOR and Psat) in the oil compared with those for the gas condensate are therefore a reflection of their lower thermal maturity and their entrapment in isolation from the gas condensate system. The GOR-Psat relationship is in agreement with the biomarker and isotope data in concluding two distinct fluid systems that are not in communication. The nature of the sealing barrier between the two fluid systems is being investigated, with a strike-slip fault (marked with a question mark in Fig. 3a) that is subparallel to the regional strike-slip fault separating Field G from the field to the north being one possibility among others^10^.

Reflection of fluid relationships and the inferred charge model discussed above in the behaviour of the phase envelopes was examined (Fig. 4b). The phase envelopes of the oil and the gas condensate form two distinct and overlapping clusters (Fig. 4b). The two types of fluids have distinct cricondentherm-cricondenbar relationships (Fig. 4c) that are controlled not only by reservoir PVT conditions, but also by charge maturity. Charging the reservoir with fluids of higher maturities decreases the molecular weight and density (hence increasing the GOR and Psat), thereby controlling the evolution of phase envelopes: shifting the cricondentherm to lower temperatures and the critical point to initially higher pressures then eventually to lower pressures (Figs. 4b and 4c). As such, the contrasting behaviour of phase envelopes provides further evidence for the accumulation of oil and gas condensate in separate systems that are not in communication, rather than in a continuous reservoir.

### Phase envelopes help identify an expansive wet gas accumulation

**Figure 5:**
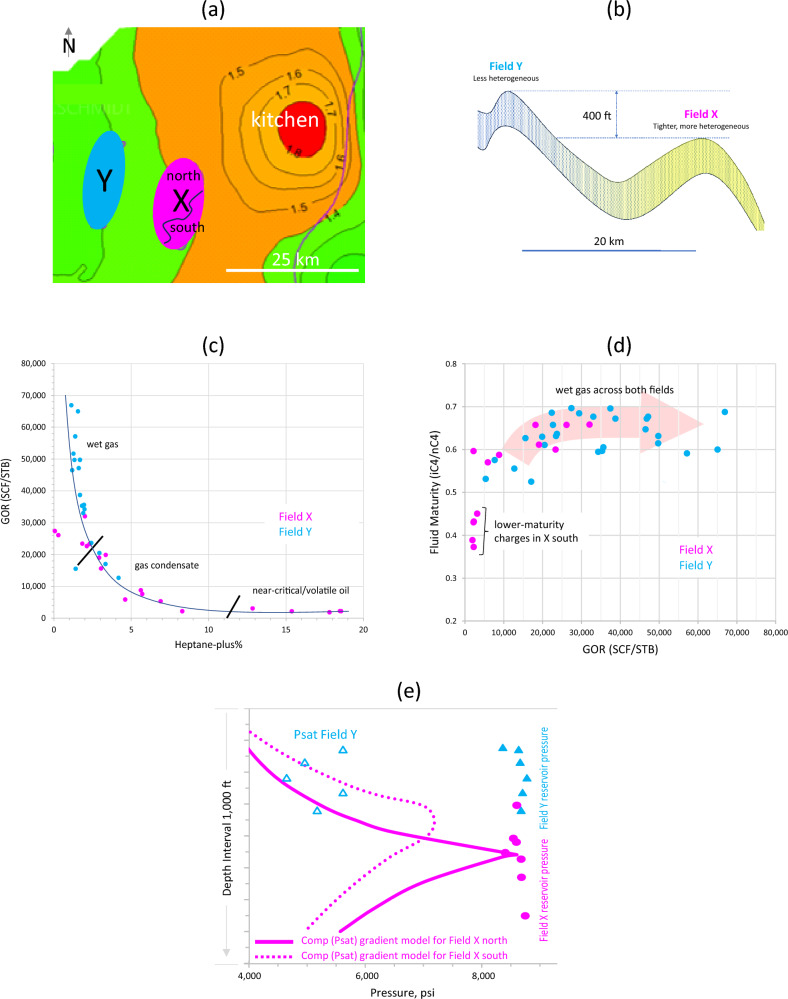
(**a**) Fields X and Y believed to be sourced from the same kitchen located to the east. (**b**) Schematic geological profile across Field X and Field Y. The sandstone reservoir is tighter and more heterogeneous in Field X than in Field Y. (**c**) Heptane-plus versus GOR relationship showing fluids evolved from mainly volatile oil/near critical and rich gas condensate in the deeper Field X to leaner gas condensate and wet gas in the shallower Field Y of up to 67,000 scf/stb. (**d**) Relationship of GOR and fluid maturity indicates that fluids in Field X resulted from charging of fluids of different maturities, while the increased GOR in Field Y is the result of further fractionation during the redistribution of wet gas across the area. (**e**) Pressure-depth profiles in Fields X and Y.

Fields X and Y are about 20 km apart and are being developed as separate gas fields (Figs. 5a and 5b). The sandstone reservoir becomes tighter and more heterogeneous towards Field X. Fluids in both accumulations are single-phased and highly undersaturated, and are believed to be sourced from the closest common kitchen located to the east (Fig. 5a). Field X plunges to the north. The downdip north of the field is mainly wet gas and rich gas condensate, grading to near critical/volatile oil towards the updip south. The 400-feet-shallower Field Y contains wet gas of up to 67,000 scf/stb (Fig. 5c). The relationship and connectivity of the different types of fluids in both fields are discussed below, with reference to their phase envelopes.

An expansive accumulation grading from volatile oil in Field X to wet gas in Field Y is suggested on the heptane-plus and GOR crossplot (Fig. 5c), a trend that is also evident in other compositional data, such as API, methane mole fraction, fluid maturity, and Psat. In addition to having the highest heptane-plus content and the lowest GOR, the volatile oil and rich gas condensate occupying the updip south of Field X are the least mature (smallest isobutane to normal butane iC4/nC4 ratios, Fig. 5d), suggesting derivation from earlier charges. Given their updip setting relative to the leaner gas in the field’s north, they appear to have accumulated by spillover from the deeper north of the field, hence forming separate compartments. The leaner gas north of Field X is more mature, a product of a more-mature charge. Despite the tripling of GOR (~ 67,000 scf/stb) in Field Y, its gas maturity is comparable to that of the wet gas in Field X (Fig. 5d). Fluids from more-mature charges normally exhibit higher GOR and higher iC4/nC4 ratios. The fact that the maturity of the shallower Field Y fluids is not increasing with the increase in GOR means that the tripling of GOR is not caused by charges of higher-maturities but the result of fractionation of the same charge during fluid redistribution and remigration. Redistribution of the wet gas within the apparently single fluid system was triggered by regional tilting towards the east, resulting in spreading the gas (that resided initially in Field X) across the area - an inference that was supported later by structural analysis^10^.

The modeled compositional (Psat) gradients for the northern and southern regions of Field X are shown in Fig. 5e. These compositional gradients represent the general (average) gradients that capture most of the compositional parameters, such as the Psat, GOR and fluid composition. Scatter is observed, which is attributed to reservoir heterogeneity that is also more evident petrophysically in the Field X area. No single Psat gradient has been established yet for Field Y, but its overall pressure gradient, Psat data and leaner gas composition appear to represent a continuation of the lean gas condensate/wet gas of the northern region of Field X (Figs. 5c-e).

**Figure 6:**
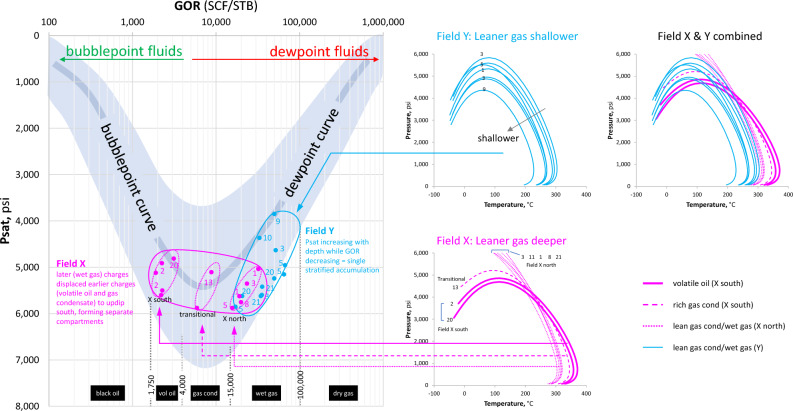
GOR-Psat relationships for fluids from Field X (purple) and Field Y (blue). The northern and southern parts of Field X constitute separate fluid regions, with a transitional composition in between, probably representing mixing. The blue band represents the global phase prediction template^13,14^. The character and evolution of phase envelopes in both fields are shown, which suggest the separation of volatile oil and gas condensate from each other in Field X, which in turn form different systems from the downdip wet gas in the same field. The wet gas in Field X grades to leaner gas in Field Y, suggesting a more-expansive accumulation linking fluids in both structures.

The separation of the wetter fluids in the southern region of Field X from the leaner fluids in its northern extension, on one hand, and the intimate relationship between the wet gas of Fields X and Y, on the other hand, are better illustrated on the GOR-Psat crossplot (Fig. 6). While the volatile oil and rich gas condensate of Field X are interpreted to belong to earlier charges locally entrapped in the southern region of the field, the wet gas appears to be common across both fields, with a systematic compositional trend suggestive of regional connectivity. The wet gas evolves/depressurises from Field X to Field Y along the dewpoint curve, giving rise to the observed gradual drop in the dewpoint pressure accompanied by the increase in GOR towards the shallower PVT conditions (Fig. 6).

The phase envelopes of representative PVT samples provide further insights into the separation or connectivity of fluids (Fig. 6). Field X is characterised by two main regions of phase envelopes representing the southern (volatile oil) and the northern (wet gas) regions of the field, with a transitional phase envelope (rich gas condensate) representing the transitional zone between the two regions. The overlapping phase envelopes for the three fluid types correspond to three different regions on the GOR-Psat crossplot and suggest derivation from different charges of variable maturities (Fig. 6). The separation between the northern and the southern regions of Field X is reflected in their unique compositional gradients, as depicted in Fig. 5d.

In contrast, Field Y fluids are characterised by systematically shrinking phase envelopes, suggestive of fractionation/segregation of a single charge across different PVT regions (Fig. 6), in agreement with the narrow maturity range discussed above (Fig. 5d). Overlying the phase envelopes of both fields clearly reveals a common behaviour for the wet gas in both fields, where the cricondentherm systematically shifts to lower temperatures and the cricondenbar to lower pressures, moving from the wet gas in Field X to the leaner gas in Field Y (Fig. 6). This uninterrupted continuity in phase evolution suggests that the gas among both fields is linked at large in a more expansive accumulation. Localised scatter is not unexpected due to variable reservoir heterogeneity. This shrinking pattern of phase envelope evolution for the wet gas is consistent with the (vertical) distribution of a single charge across regions of varying PVT conditions, from the deeper Field X to the shallower Field Y. Shallowing-up phase fractionation/segregation reduces the density and molecular weight of the gas phase, hence increasing the GOR while decreasing the Psat along the dewpoint trend, and systematically shrinking the phase envelopes to lower temperatures and pressures.

## Conclusions

Two contrasting scenarios were presented to demonstrate criteria and value of phase envelopes for reservoir fill analysis, particularly when constrained with fluid geochemistry and available PVT data. The first discussed an oil-rimmed gas accumulation (Field G), which, based on employing phase envelopes, in addition to other geochemical characteristics, was found to be otherwise; i.e., compartmentalised into separate oil and gas regions from separate charges. The second example presented an opposite scenario where two wet gas accumulations (Fields X and Y) with an interrelated phase evolution appear to belong to a single more-expansive accumulation spread over a wide area. The proposed gas distribution model combining both Fields X and Y into a single more-expansive gas accumulation has helped optimise field development and will support prospecting for additional gas resources. This type of analysis is highly recommended for petroleum exploration and optimisation of field development.

## Data Availability

The datasets used and/or analysed during the current study are available from the corresponding author on reasonable request.
